# Alterations in erythrocyte fatty acid composition in preclinical Alzheimer’s disease

**DOI:** 10.1038/s41598-017-00751-2

**Published:** 2017-04-06

**Authors:** Kathryn Goozee, Pratishtha Chatterjee, Ian James, Kaikai Shen, Hamid R. Sohrabi, Prita R. Asih, Preeti Dave, Bethany Ball, Candice ManYan, Kevin Taddei, Roger Chung, Manohar L. Garg, Ralph N. Martins

**Affiliations:** 1 Anglicare, Sydney, NSW Australia; 2 School of Psychiatry and Clinical Neurosciences, University of Western Australia, Crawley, WA Australia; 3 School of Medical and Health Sciences, Edith Cowan University, Joondalup, WA Australia; 4 Department of Biomedical Sciences, Macquarie University, North Ryde, NSW Australia; 5 KaRa Institute of Neurological Diseases, Macquarie Park, NSW Australia; 6 McCusker Alzheimer Research Foundation, Nedlands, WA Australia; 7 The Cooperative Research Centre for Mental Health, Carlton, VIC Australia; 8 Institute for Immunology & Infectious Diseases, Murdoch University, Murdoch, WA Australia; 9 Australian eHealth Research Centre, CSIRO, Australia; 10 School of Medical Sciences, University of New South Wales, Kensington, NSW Australia; 11 Nutraceuticals Research Program, School of Biomedical Sciences and Pharmacy, University of Newcastle, Callaghan, NSW Australia

## Abstract

Brain and blood fatty acids (FA) are altered in Alzheimer’s disease and cognitively impaired individuals, however, FA alterations in the preclinical phase, prior to cognitive impairment have not been investigated previously. The current study therefore evaluated erythrocyte FA in cognitively normal elderly participants aged 65–90 years via trans-methylation followed by gas chromatography. The neocortical beta-amyloid load (NAL) measured via positron emission tomography (PET) using ligand ^18^F-Florbetaben, was employed to categorise participants as low NAL (standard uptake value ratio; SUVR < 1.35, N = 65) and high NAL or preclinical AD (SUVR ≥ 1.35, N = 35) wherein, linear models were employed to compare FA compositions between the two groups. Increased arachidonic acid (AA, p < 0.05) and decreased docosapentaenoic acid (DPA, p < 0.05) were observed in high NAL. To differentiate low from high NAL, the area under the curve (AUC) generated from a ‘base model’ comprising age, gender, *APOE*ε4 and education (AUC = 0.794) was outperformed by base model + AA:DPA (AUC = 0.836). Our findings suggest that specific alterations in erythrocyte FA composition occur very early in the disease pathogenic trajectory, prior to cognitive impairment. As erythrocyte FA levels are reflective of tissue FA, these alterations may provide insight into the pathogenic mechanism(s) of the disease and may highlight potential early diagnostic markers and therapeutic targets.

## Introduction

Alzheimer’s disease (AD), a progressive neurodegenerative disorder, is the most common form of dementia. While it is estimated that 47 million people worldwide are presently living with dementia, it has been projected to escalate to 76 million by 2030. Currently there is no definitive diagnosis or effective treatment for AD. Understanding the molecular changes occurring from the preclinical stage of the disease, may present a better chance to ameliorate AD related pathogenic changes prior to them becoming irreversible.

Given that fatty acids are essential components of cell membranes, playing a vital role in normal cellular functioning, several studies have reported altered brain fatty acid composition in AD^1–3^. Substantial evidence also presents an association between altered blood fatty acid concentrations and, cognitive impairment and dementia risk, particularly to that of AD^4–6^. Further, an increasing number of studies suggest that omega-3 (n-3) polyunsaturated fatty acids (PUFA), primarily docosahexaenoic acid (DHA) and eicosapentaenoic acid (EPA) are protective against cognitive impairment and dementia^7–11^, whereas arachidonic acid (AA), an omega-6 (n-6) PUFA, contributes towards AD pathogenesis^12–15^.

The protective and deleterious effects of fatty acids have been explored with respect to cognitive impairment and clinical AD, however alterations in fatty acid profiles in individuals within the preclinical stage of AD, prior to any apparent cognitive impairment, have not been investigated before.

Since aberrant accumulation of neocortical beta-amyloid load (NAL) has been reported to begin 15–20 years prior to the clinical manifestation of AD^16^, therefore serving as a preclinical feature of the disease, we compared the erythrocyte fatty acid composition of cognitively normal participants with high NAL (Standard uptake value ratio or SUVR ≥ 1.35) versus those with low NAL (SUVR < 1.35), to evaluate alterations in fatty acid metabolism that manifest in the periphery, in the asymptomatic preclinical phase of AD, within this exploratory study.

## Results

### Cohort characteristics

Participant demographics, serum cholesterol and hormone levels, *APOE*ε4 carrier status, MMSE scores and neuroimaging data including NAL and hippocampal volume have been presented in Table 1.

**Table 1:** Characteristics of study participants.

	Low NAL	High NAL	p
Gender (M/F)	19/46	13/22	0.419
Age (years, mean ± SD)	77.61 ± 5.55	79.22 ± 5.38	0.165
BMI (mean ± SD)	27.38 ± 4.47	28.05 ± 4.73	0.486
Education (years, mean ± SD)	14.86 ± 3.31	13.66 ± 2.84	0.078
Total cholesterol (mmol/L, mean ± SD)	4.82 ± 0.98	4.35 ± 1.19	0.045
Triglycerides (mmol/L, mean ± SD)	1.34 ± 0.70	1.10 ± 0.42	0.071
HDL cholesterol (mmol/L, mean ± SD)	1.64 ± 0.55	1.52 ± 0.48	0.292
LDL cholesterol (mmol/L, mean ± SD)	2.39 ± 0.85	2.34 ± 0.88	0.794
Testosterone (nmol/L, mean ± SD), males	14.43 ± 6.16	11.66 ± 3.77	0.160
Testosterone (nmol/L, mean ± SD), females	1.45 ± 2.55	1.26 ± 0.81	0.738
Oestradiol (pmol/L, mean ± SD), males	113.16 ± 47.83	117.23 ± 35.17	0.795
Oestradiol (pmol/L, mean ± SD), females	91.22 ± 118.71	73.90 ± 28.97	0.523
*APOE ε4* carriers (%)	7.69	45.71	0.00000848
MMSE (mean ± SD)	28.50 ± 1.16	28.80 ± 1.10	0.225
FBB-PET SUVR (mean ± SD)	1.15 ± 0.08	1.71 ± 0.26	—
HV% (left; right lobes, mean ± SD)	0.195 ± 0.020; 0.199 ± 0.021	0.194 ± 0.019; 0.199 ± 0.018	0.805; 0.890

Baseline characteristics including gender, age, body mass index (BMI), education, serum cholesterol, hormone levels, *APOE ε4* status, mini mental state examination (MMSE) scores, neocortical amyloid load (NAL) represented by the standard uptake value ratio (SUVR) of the ligand ^18^F-Florbetaben (FBB) in the neocortical region normalised with that in the cerebellum and hippocampal volume (HV) normalised by the intracranial volume, have been compared between study participants with low NAL (SUVR < 1.35) and high NAL (SUVR ≥ 1.35). Chi-square test or linear models were employed as appropriate. HDL: high density lipoprotein, LDL: low density lipoprotein.

All participants had a MMSE score ≥26, indicating absence of cognitive impairment. While no significant differences in demographic data, hormone levels, MMSE and hippocampal volume were observed between low and high NAL, the frequency of *APOE*ε4 carriers was significantly higher in participants with high NAL compared to those with low NAL (p < 0.0005).

### Erythrocyte fatty acids and demographic characteristics

Among the erythrocyte FA measured, linoleic acid (C18:2n-6, β = −0.228, p = 0.022) and DHA (C22:6n-3, β = −0.184, p = 0.066) were observed to be inversely associated with age (Supplementary Table S1).

Further, myristic acid (C14:0; p < 0.01), arachidic acid (C20:0; p < 0.05) and DHA (p < 0.05) were observed to be significantly lower in males (n = 32) compared to females (n = 68), while DPA (C22:5n-3; p < 0.01) was higher in males compared to females with and without adjusting for appropriate covariates. Furthermore, after adjusting for covariates, palmitoleic acid (C16:1; p < 0.05) was significantly higher in females compared to males (Supplementary Table S2).

### Erythrocyte fatty acids in *APOE*ε4 carriers and memory complainers

On comparing erythrocyte FA measured between *APOE*ε4 non-carriers (n = 79) and carriers (n = 21), linoleic acid (C18:2n-6; p < 0.01) and eicosadienoic acid (C20:2n-6; p < 0.05) were observed to be higher in the non-carriers with and without adjusting for appropriate covariates. Additionally, dihomo-γ-linolenic acid also appeared to be higher in the non-carriers after adjusting for appropriate covariates (Supplementary Table S3).

Further, categorising participants into subjective memory complainers (n = 76) and non-complainers (n = 24), myristic acid was observed to be significantly higher (p = 0.006) in memory complainers compared to non-complainers, with and without adjusting for covariates age, gender, *APOE*ε4, years of education and NAL (Supplementary Table S4).

### Erythrocyte fatty acids and neocortical beta-amyloid load

**Figure 1:**
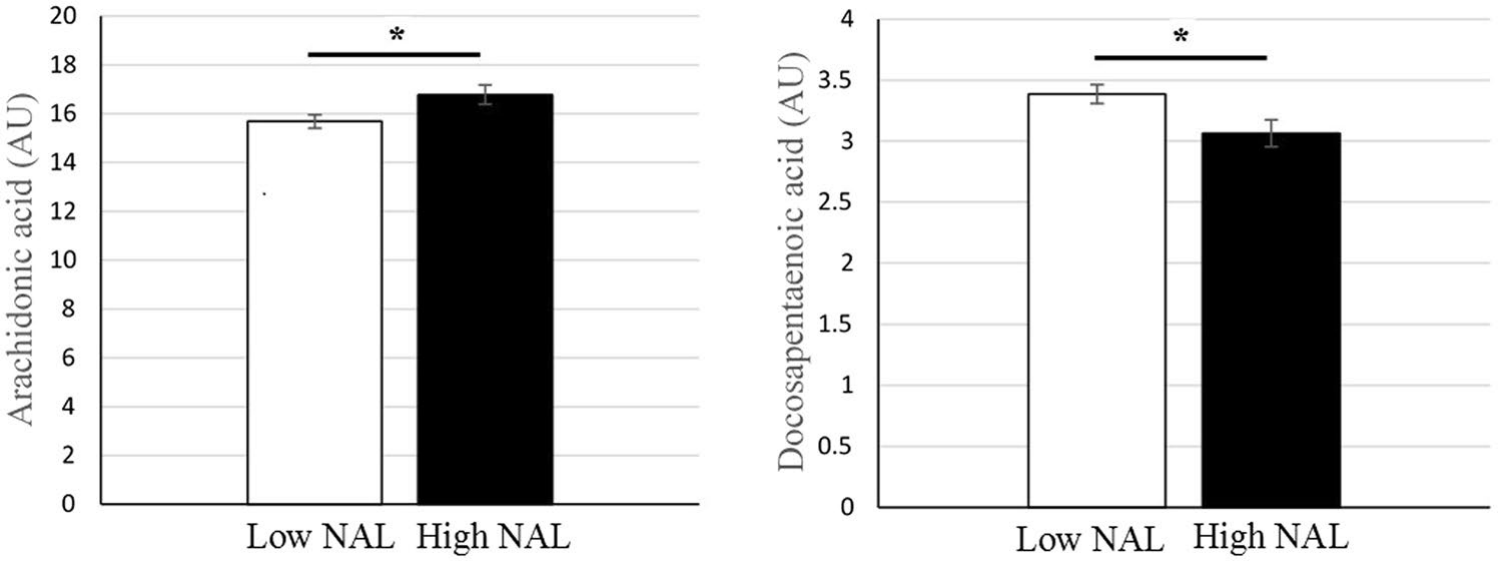
Altered fatty acid levels in cognitively normal individuals with low and high NAL. Elevated erythrocyte arachidonic acid levels and decreased docosapentaenoic acid (n-3) levels were observed in individuals with high NAL (N = 35) compared to those with low NAL (N = 65), based on standard uptake value ratio cut off score of 1.35. Fatty acid concentrations were measured in arbitrary units (AU). ‘*’ represents p < 0.05, adjusted for covariates age, gender, years of education and *APOE ε4* status; NAL: neocortical amyloid load measured via positron emission tomography, using ligand ^18^F-Florbetaben. The error bars represent SE.

When erythrocyte FA measured in the current study were compared between low and high NAL, no significant differences were observed in saturated fatty acids (SFA) and mono unsaturated fatty acids (MUFA) between the two groups, with and without adjusting for covariates. Within the n-6 PUFA measured, linoleic acid (C18:2n-6; p < 0.05) was significantly lower and AA (p < 0.05) significantly elevated in high NAL compared to low NAL; however, after adjusting for covariates, only AA remained significant (p < 0.05). Among the n-3 PUFA measured, linolenic acid (C18:3n-3) was significantly lower in high NAL (p < 0.05), however after adjusting for covariates the significance in linolenic acid disappeared while docosapentaenoic acid (DPA, C22:5n-3, p < 0.05) was observed to be significantly lower in high NAL (Fig. 1). Additionally, a trend of lower EPA concentrations (p = 0.063) and higher stearic acid levels (p = 0.062) was observed in high NAL compared to low NAL (Table 2).

**Table 2:** Erythrocyte fatty acid concentrations in low and high NAL.

	Low NAL (mean ± SD)	High NAL (mean ± SD)	p	p^a^	p^b^
***SFA***					
C14:0 (Myristic acid)	0.40 ± 0.07	0.38 ± 0.06	0.184	0.335	0.364
C16:0 (Palmitic acid)	21.94 ± 1.40	21.67 ± 0.85	†0.374	†0.576	†0.610
C18:0 (Stearic acid)	15.70 ± 1.03	16.05 ± 0.80	0.088	0.062	0.080
C20:0 (Arachidic acid)	0.40 ± 0.07	0.42 ± 0.06	0.196	0.128	0.157
C24:0 (Lignoceric acid)	5.03 ± 2.29	4.77 ± 0.84	0.520	0.217	0.236
Total SFA	43.49 ± 0.87	43.31 ± 1.21	0.392	0.157	0.157
***MUFA***					
C14:1 (Myristoleic acid)	0.01 ± 0.00	0.01 ± 0.01	0.171	0.306	0.313
C16:1 (Palmitoleic acid)	0.34 ± 0.13	0.30 ± 0.08	†0.345	†0.265	†0.239
C18:1n-9 (Oleic acid)	8.74 ± 0.71	8.77 ± 0.64	0.824	0.946	0.887
C18:1n-7 (Vaccenic acid)	1.91 ± 0.43	2.02 ± 0.61	†0.385	†0.865	†0.787
C20:1n-9 (Eicosenoic acid)	0.25 ± 0.05	0.26 ± 0.07	†0.578	†0.231	†0.246
C24:1 (Nervonic acid)	4.92 ± 0.92	5.06 ± 0.89	0.478	0.841	0.831
Total MUFA	16.19 ± 1.25	16.45 ± 1.38	0.334	0.939	0.921
***n***-***6 PUFA***					
C18:2n-6 (Linoleic acid)	8.82 ± 1.41	8.23 ± 1.48	0.052	0.878	0.924
C18:3n-6 (γ-Linolenic acid)	0.77 ± 0.73	1.08 ± 1.64	†0.928	†0.244	†0.281
C20:2n-6 (Eicosadienoic acid)	0.14 ± 0.04	0.13 ± 0.04	0.300	0.717	0.668
C20:3n-6 (Dihomo-γ-linolenic acid)	1.60 ± 0.46	1.66 ± 0.41	0.586	0.237	0.301
C20:4n-6 (Arachidonic acid)	15.70 ± 2.12	16.72 ± 1.91	**0.020**	**0.035**	**0.043**
Total omega-6	27.07 ± 2.62	27.84 ± 2.29	0.145	**0.023**	**0.027**
***n***-***3 PUFA***					
C18:3n-3 (Linolenic acid)	0.16 ± 0.10	0.12 ± 0.07	**0.033**	0.076	0.084
C20:5n-3 (Eicosapentaenoic acid)	1.72 ± 0.96	1.42 ± 0.69	0.106	0.063	0.068
C22:5n-3 (Docosapentaenoic acid)	3.33 ± 0.64	3.15 ± 0.50	0.143	**0.027**	**0.025**
C22:6n-3 (Docosahexaenoic acid)	8.01 ± 1.59	7.68 ± 1.40	0.308	0.458	0.512
Total omega-3	13.24 ± 2.72	12.38 ± 2.20	0.111	0.100	0.116
Omega-3 Index	9.73 ± 2.32	9.10 ± 1.96	0.176	0.337	0.233

Using linear models, fatty acid concentrations were compared between study participants with low (N = 65) and high (N = 35) neocortical amyloid load (NAL) represented by the standard uptake value ratio (SUVR) of the ligand ^18^F-Florbetaben in the neocortical region normalised with that in the cerebellum. Low NAL was defined as SUVR < 1.35 while high NAL was defined as SUVR ≥ 1.35. p^a^ indicates p values adjusted for age, gender, years of education and *APOE ε4* status; p^b^ indicates p values adjusted age, gender, years of education, *APOE ε4* status, body mass index, fatty acid supplement intake (cod liver oil, flaxseed oil), hormone replacement therapy. ‘†’ indicates p-values obtained from variables transformed to the logarithmic scale for analyses. SFA: saturated fatty acids; MUFA: monounsaturated fatty acids; PUFA: polyunsaturated fatty acids; omega-3 index: sum of eicosapentaenoic acid and docosahexaenoic acid, expressed as a percentage of total erythrocyte fatty acid measured.

On investigating associations between NAL and erythrocyte fatty acids measured, a positive trend between AA concentrations and NAL (β = 0.197, p = 0.050) was observed, while an inverse trend was seen for linoleic acid (β = −0.172, p = 0.088).

### Erythrocyte fatty acids and hippocampal volume

Further an inverse though non-significant association was observed between oleic acid and the left hippocampal volume (β = −0.184, p = 0.073), while DHA (β = 0.170, p = 0.098) was seen to have a positive though non-significant association with the left hippocampal volume. Interestingly, within the subjective memory complainer group, these associations between hippocampal volume and oleic acid (β = −0.264, p = 0.023), and DHA (β = 0.229, p = 0.050) became stronger.

### Dietary intake of arachidonic acid and docosapentaenoic acid

Further, to explore whether dietary FA intake directly attributed to the alterations we observed in erythrocyte fatty acid concentrations, we assessed the dietary intake of fatty acids that were observed to be significantly different in erythrocytes of participants with high NAL against those with low NAL (primarily AA, DPA) and between *APOE*ε4 carriers and non-carriers (linoleic acid, eicosadienoic acid, dihomo-γ-linolenic acid), employing data from the CCVFFQ. No significant difference was observed in the dietary intake of AA and DPA between high NAL and low NAL with and without adjusting for covariates age, BMI, years of education and gender (Supplementary Table S5). Further, while lower dietary intake of eicosadienoic acid and dihomo-γ-linolenic acid were noted based on data from the CCVFFQ in *APOE*ε4 carriers compared to the non-carriers with and without adjusting for covariates age, BMI, years of education and gender, no significant difference in linoleic acid dietary intake was observed between *APOE*ε4 carriers and non-carriers (Supplementary Table S5).

### Ratio of AA:DPA as a biomarker for predicting high neocortical beta-amyloid load

**Figure 2:**
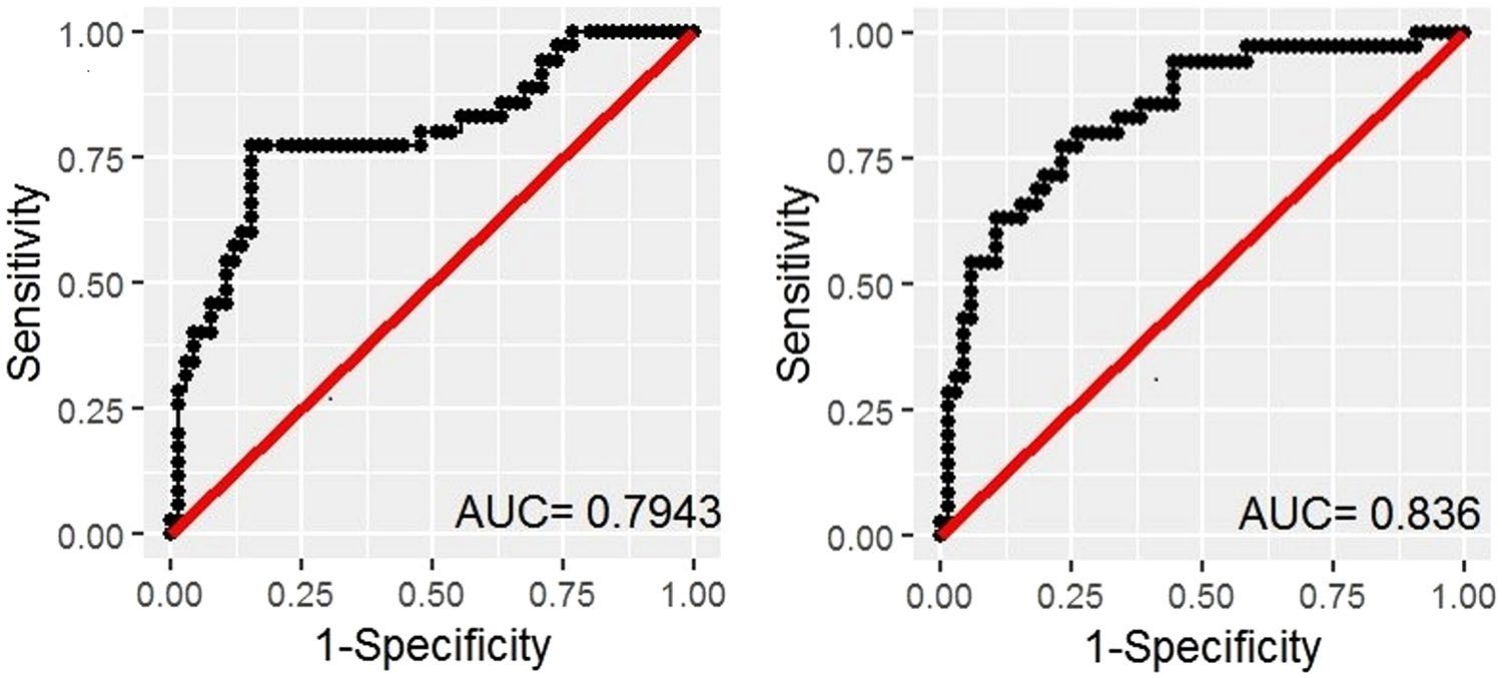
Receiver operating characteristic curves for the prediction of high neocortical amyloid load. Receiver operating characteristic curves of logistic regression modelling shows that the specificity of the ‘base’ model comprising major risk factors age and *APOE ε4* allele status, and gender and education (**a**) was enhanced by adding the ratio of AA:DPA i.e. ‘base + AA:DPA’ model (**b**) at 80% sensitivity. AUC: area under the curve; AA: arachidonic acid, DPA: Docosapentaenoic acid n-3.

Finally, given the observations of higher AA and lower DPA concentrations in high NAL compared to low NAL, we also evaluated the ratio of AA:DPA as a potential biomarker, by generating a ‘base’ model comprising age, gender, *APOE*ε4 allele status and education, and compared it with ‘base + AA:DPA’ model, wherein the ratio of AA:DPA was added to the ‘base’ model. After adjusting for age, gender, *APOE*ε4 allele status and education, the ratio of AA:DPA was significantly associated with low and high NAL (p = 0.02). The receiver operating characteristic (ROC) curves for the above mentioned models have been presented in Fig. 2, such that the AUC of the ‘base + AA:DPA’ model (AUC = 0.836, 80% sensitivity, 73.8% specificity) was observed to outperform the ‘base’ model (AUC = 0.794, 80% sensitivity, 52.3% specificity).

## Discussion

The current study reports erythrocyte fatty acid alterations, primarily in, AA and DPA, in elderly individuals with high NAL against those with low NAL, prior to cognitive impairment and significant differences in hippocampal atrophy (Table 1).

Elevated levels of erythrocyte AA were observed in high NAL, a feature of preclinical AD, compared to low NAL. Interestingly, several studies provide evidence to support the contributory role of increased AA to AD pathogenesis^12–14, 17–19^. AA and its pro-inflammatory eicosanoid metabolites such as prostaglandins, thromboxanes, and leukotrienes have been reported to promote beta-amyloid secretion^13^ and therefore not surprisingly AA metabolism is increased in AD brains^12, 14^. Furthermore, the depletion of an enzyme responsible for the release of AA (cytosolic phospholipase A2), has been reported to be protective against deficits in memory and learning in a transgenic AD mouse model^17^. AA has been shown to trigger apoptotic pathways in neurons^20^ and *in vitro* studies further suggest that AA promotes tau polymerization^18, 19^. The above studies consistently demonstrate that AA is positively associated with AD neuropathology and our findings, further show that an elevation in AA in the blood at a preclinical stage reflects preclinical-AD related pathogenic changes in the brain.

On the other hand, a decline in erythrocyte DPA (n-3) levels, an intermediate between EPA and DHA, was observed in high NAL participants compared to low NAL. Given that AA and DPA (n-3) compete for the elongase enzyme^21^, it may be posited that elevated AA levels contribute to the declining levels of DPA (n-3) in high NAL. Interestingly, decreased erythrocyte DPA (n-3) concentrations have been reported in another neurological condition; patients with schizophrenia were observed to have 22% lower erythrocyte DPA (n-3) concentrations compared to healthy controls^22^. Conversely, DPA (n-3) supplementation has been observed to have neuroprotective and restorative effects on neuronal function in aged rats which acts through its ability to decrease age related oxidative stress and microglial activation^23^, therefore highlighting the anti-oxidative and anti-inflammatory properties of DPA.

DPA (n-3) levels were observed to be significantly lower in females compared to males. We therefore investigated DPA (n-3) levels after categorising participants based on gender and while we continued to find a significant decline in DPA (n-3) concentrations in high NAL males compared to low NAL males (p < 0.05) with and without adjusting for covariates, this significance disappeared in females; however, this could be attributed to the modest sample size available for the observed effect size.

However, in contrast to the significant associations often reported between lower blood DHA (and EPA) concentrations and increased risk to AD in participants with early symptomatic^24^ or clinical dementia^6, 10, 11^, significant alterations in erythrocyte DHA levels were not apparent between high and low NAL participants within the current study wherein all participants were cognitively normal. Given that previous findings suggest decreased blood DHA and EPA concentrations are linked with cognitive decline or impairment^6, 25^, may explain the absence of significantly declined erythrocyte DHA and EPA observations in high NAL compared to low NAL within the present study which only comprised participants who were within the cognitively normal range; however, this observation may also be attributed to the modest sample size employed within the study. Investigating whether participants falling within the lowest quartile of DHA at baseline (time point used in current study), become symptomatic at their one year follow-up visit, will provide further insight to our current report. However, the current findings indicate that alterations in AA and DPA manifest in the asymptomatic stage of the disease, and are not a direct result of dietary intake, and therefore may shed light on early disease mechanisms that may serve as beneficial therapeutic targets.

Additionally, an inverse association between oleic acid and hippocampal volume was observed; given that hippocampal atrophy is a pathological hallmark of AD, and that elevated oleic acid concentrations feature in the more advanced stages of AD pathogenesis^26^ our findings in subjective memory complainers indicate that oleic acid may be an early marker for hippocampal atrophy.

DHA has also been shown to be associated with hippocampal volume in early symptomatic dementia^24^ and clinical AD wherein participants with erythrocyte DHA levels in the first quartile, had lower total brain volumes compared to the upper 3 quartiles^27^. Similar to findings in our study, a trend of association between DHA and hippocampal volume has been highlighted previously by Pottala *et al*. in an elderly cohort that was free of dementia^28^. Furthermore, on categorizing participants into quartiles based on their erythrocyte DHA levels as employed by Tan *et al*., we also observed significantly higher hippocampal volume in participants with erythrocyte DHA levels in quartile 3 (mean ± SD: 0.20 ± 0.02) compared to quartile 1 (mean ± SD: 0.18 ± 0.01, p = 0.002) and a trend in quartiles 2 (mean ± SD: 0.19 ± 0.01, p = 0.068) and 4 (mean ± SD: 0.19 ± 0.01, p = 0.058) compared to quartile 1.

In addition, since the ε4 allele of *APOE* is a well-established risk factor for AD, we also investigated FA differences between non-carriers and carriers of the ε4 allele. Interestingly, significantly lower n-3 PUFA linolenic acid, and n-6 PUFA, linoleic acid and eicosadienoic acid, were observed in the *APOE*ε4 carriers compared to the non-carriers. These differences in linoleic acid and eicosadienoic acid remained significant following adjustment for covariates NAL, age, gender, BMI, education, supplement intake and hormone replacement therapy. Given that the dietary intake of linoleic acid from the CCVFFQ data did not indicate differential dietary intake between the two groups, it may be posited that an altered metabolism of linoleic acid occurs in *APOE*ε4 carriers^29^. Additionally, lower linoleic acid concentrations in *APOE*ε4 carriers along with the inverse trend observed between linoleic acid and NAL, may suggest that decreased linoleic acid may be indicative of an increased risk to AD; this decreased level of linoleic acid could be a result of increased metabolism of linoleic acid to arachidonic acid and its metabolites. Our findings in *APOE*ε4 carriers together with several other studies^29–33^, indicate the presence of a differential responsiveness to PUFA metabolism.

The erythrocyte FA alterations observed between males and females, and between memory complainers and non-complainers, did not appear to be traceable to their dietary intake as assessed from the CCVFFQ (Supplementary Table S5). However, the authors acknowledge that data from food frequency questionnaires do not necessarily reflect an accurate evaluation of the dietary intake of FA but rather serve as a suitable reflection of exposure to certain food and nutrient types over a period of time, compared to the erythrocyte FA composition that reflects fatty acid intake or metabolism of approximately 120 days.

To conclude, while it is acknowledged that the current study employs a modest sample size and that further longitudinal studies with larger sample sizes are required to validate the current novel observations, findings from the present study may imply that elevated AA and decreased DPA erythrocyte concentrations are an early event in the AD pathogenesis trajectory. Further, several studies have presented fatty acid alterations associated with cognitive decline and clinical AD, however the current study, using PET beta-amyloid imaging, is to our knowledge, the first to report fatty acid alterations associated with the preclinical phase of AD prior to cognitive impairment and disease related neuronal atrophy. In addition to highlighting the need for a re-evaluation of AA supplementation as an early therapeutic approach for AD^34, 35^, given the urgent need for early diagnosis and intervention strategies, our findings also provide insight on a potential early diagnostic candidate as well as alternative preventative targets for AD. Our findings of elevated AA and decreased DPA in subjects with high NAL indicate that inflammation and oxidative stress are early features of preclinical AD, and highlights the importance of evaluating anti-inflammatory and anti-oxidative approaches to combat AD pathogenesis at its earliest.

## Methods

### Participants

Participants belonged to the McCusker Kerr Anglican Retirement Village Initiative in Ageing Health (KARVIAH) study cohort, at baseline. Participants were residents of Anglicare (formally known as Anglican Retirement Villages), aged 65–90 years, living within retirement living accommodations in Sydney, Australia. Among the 206 volunteers, 143 met the set inclusion and exclusion criteria (refer to supplementary material) and were deemed eligible for the KARVIAH cohort. One hundred and five participants, out of the 143 volunteers meeting the inclusion and exclusion criteria, at the screening phase for this study, progressed to undertake neuropsychological testing, blood collection and neuroimaging, since the remaining either withdrew from the study or declined undergoing positron emission tomography.

Following the screening assessment, among the 105 participants, 100 participants were recorded to have a Mini Mental State Examination (MMSE) score ≥26 at baseline, and were therefore selected for the current study. Within these 100 participants (of which four reported oral fatty acid supplement intake and two participants were undergoing hormone replacement therapy), 65 were categorised as low NAL and 35 as high NAL, based on their neocortical beta-amyloid load assessed via positron emission tomography (PET). Written informed consent was obtained from all participants. The Bellberry Human Research Ethics Committee Australia provided approval for the study and all methods were performed in accordance with the relevant guidelines and regulations.

### Physical examination and sample processing

Overnight fasted participants underwent a brief physical examination measuring weight, height, pulse and blood pressure, followed by blood draw. Subsequently, erythrocytes were isolated from the blood collected and fractionated as previously described^36^ and were stored at −80 °C, until used for further analyses. Apolipoprotein E (*APOE*) genotype was determined from 0.5 ml whole blood employing standard PCR methodology^37^.

### Erythrocyte fatty acid analysis

#### Fatty acid derivatisation and gas chromatography

Erythrocyte samples underwent transesterification prior to gas chromatography^38^. Briefly, 2 ml of methanol-toluene (4:1, v/v) containing 20 µg/ml of C19:0 as internal standard was added to 200 µl of the thawed sample, followed by the addition of 200 ul acetyl chloride while vortexing, after which the tubes were heated for 1 hour at 100 °C. The tubes were then cooled in water for 5 min following which 5 ml of 6% K_2_CO_3_ was added to the tubes prior to centrifugation at 3000 g for 5 min at 4 °C. The upper toluene phase was collected and stored in a gas chromatography (GC) vial at −20 °C until GC analysis was performed.

Fatty acid methyl esters were analysed by gas chromatography (Shimadzu GC-2010 Plus system) using a 30 m × 0.25 mm ID Restek Famewax (Shimadzu Scientific) fused silica capillary column. Ultra-pure hydrogen was used as a carrier and the oven temperature was programmed from 130 °C to 225 °C at a rate of 6 °C per min. Injector and detector temperatures were set at 220 °C and 230 °C, respectively. A split ratio of 1:30 and an injection volume of 1 µl were used. Internal standard methylnonadecanoate (19:0; cat. no. N-5377, Sigma-Aldrich) and a fatty acid mixture (cat. no. 18919-1AMP SUPELCO, Sigma-Aldrich) were used to compare analysed samples to identify peaks according to retention time. Calibration curves for each fatty acid were built using a range of concentrations of a mixture of authentic fatty acid standards. Chromatography data were recorded and integrated using LabSolutions software (Version 5.81 SP1). Individual fatty acid concentrations were reported as a percentage of total erythrocyte fatty acids measured.

### Dietary fatty acid intake

The dietary intake of specific erythrocyte FA observed to be altered between high and low NAL, and *APOE*ε4 carriers and non-carriers, were investigated to establish whether diet attributed to the observations made, employing data from the Cancer Council of Victoria Food Frequency Questionnaire (CCVFFQ)^39^. The CCVFFQ is a paper-based semi-quantitative seventy-four item questionnaire. The CCVFFQ was optically scanned to obtain nutrient intakes in gram/day. Participants filled out the CCVFFQ when they attended the research centre for their baseline assessment.

### Neuropsychological tests

Study participants underwent a comprehensive battery of neuropsychological tests, measuring episodic memory, executive function, attention, visuospatial abilities, language, processing speed and working memory, however, for the current study only MMSE (range 0–30, indicating severe impairment to no impairment) scores^40^ were employed to confirm no cognitive impairment, and the Memory Assessment Clinic - Questionnaire (MAC-Q) scores^41^ were considered to differentiate between subjective memory complainers (range 25–35) from non-complainers (range ≤24).

### Neuroimaging

All participants within the current study underwent PET imaging using ligand ^18^F-Florbetaben (FBB) and magnetic resonance imaging (MRI) within three months of blood collection, at the Macquarie Medical Imaging located within Macquarie University Hospital, Sydney, Australia. Participants were administered an intravenous bolus of FBB slowly over 30 s, while in a rested position. Images were acquired over a 20 min scan beginning 50 min post injection. NAL was calculated as the mean standard uptake value ratio (SUVR) of the frontal, superior parietal, lateral temporal, lateral occipital, and anterior and posterior cingulate^42, 43^. An SUVR cut-off value of 1.35 was utilised to categorise participants into high and low NAL.

In addition, participants underwent a sagittal 3D T1 spoiled gradient recalled acquisition in steady state and 3D T2 fluid-attenuated inversion recovery with axial dual echo Turbo spin echo PD/T2 weighted sequences and axial T2 star gradient sequence scans, performed on a General Electric 3 Tesla scanner (GE, Model 750 W), utilising a 24 channel head coil. Hippocampal volume was calculated from the images acquired and was normalized by dividing with the total intracranial volume consisting of the sum of the cerebrospinal fluid, gray matter and white matter volumes. Due to MRI exclusion criteria and related health conditions, 4 of the 100 were unable to undergo the MRI imaging component of the study.

### Statistical Analyses

Descriptive statistics were calculated for low and high NAL groups. Chi-square tests were employed to compare gender and *APOE*ε4 carrier status between low and high NAL groups. Linear models were utilised to compare continuous parameters between groups of interest, with and without adjusting for covariates age, gender, *APOE*ε4, education, body mass index, supplement intake and hormone replacement therapy, and to investigate associations between fatty acid measures and age and, neuroimaging data. All continuous parameters were checked for normality, and those that deviated were transformed appropriately prior to conducting analyses. The receiver operator characteristic (ROC) curves and the area under the curve (AUC) were derived from the predictive probabilities of the logistic regression models. No correction for multiple comparisons was made, due to the exploratory nature of the current study. All analyses were carried out using IBM^®^ SPSS^®^ Version 20, except for the ROC curves, which were generated using package, Deducer on R (version 3.2.5).

## Electronic supplementary material

Supplementary material
