# Supplementary material for: Alterations in erythrocyte fatty acid composition in preclinical Alzheimer’s disease
Source: Sci Rep. 2017 Apr 6;7:676. doi: 10.1038/s41598-017-00751-2 (PMC5429676; doi:10.1038/s41598-017-00751-2)
Supplement: Supplementary file 1 — Supplementary material [file 41598_2017_751_MOESM1_ESM.pdf]

Alterations in erythrocyte fatty acid composition in preclinical Alzheimer's disease

Kathryn Goozee [ǂ], Pratishtha Chatterjee [ǂ], Ian James, Kaikai Shen, Hamid R. Sohrabi, Prita R. Asih, Preeti Dave, Bethany Ball, Candice ManYan, Kevin Taddei, Roger Chung, Manohar L. Garg, Ralph N. Martins*

Supplementary material

The Kerr Anglican Retirement Village Initiative in Ageing Health (KARVIAH) cohort inclusion and exclusion criteria relevant to the current study constituted:

Inclusion criteria: Age 65-90 years with good health and no history of significant cerebral vascular disease, fluent in English, adequate vision and hearing to enable testing, normal general cognitive function as determined by Montreal Cognitive Assessment (MoCA) [1] scores greater than or equal to 26 while individuals with MoCA scores ranging between 23-25 (n=22) were assessed by a neuropsychologist, whereby scores were stratified according to age and education.

Exclusion criteria: Diagnosis of dementia based on the revised criteria from the National Institute on Aging - Alzheimer's Association, presence of acute functional psychiatric disorder (including lifetime history of schizophrenia or bipolar disorder), history of stroke, presenting with depression based on the Depression, Anxiety, Stress Scales (DASS) and uncontrolled hypertension (systolic BP > 170 or diastolic BP > 100).

Supplementary table S1. Associations between erythrocyte fatty acid concentrations and age.

	**β**	**p**
**SFA**		
C14:0 (Myristic acid)	.114	NS
C16:0 (Palmitic acid)	.081	NS
C18:0 (Stearic acid)	-.144	NS
C20:0 (Arachidic acid)	-.118	NS
C24:0 (Lignoceric acid)	.136	NS
**MUFA**		
C14:1 (Myristoleic acid)	-.020	NS
C16:1 (Palmitoleic acid)	.126	NS
C18:1n-9 (Oleic acid)	.058	NS
C18:1n-7 (Vaccenic acid)	.116	NS
C20:1n-9 (Eicosenoic acid)	.061	NS
C24:1 (Nervonic acid)	.060	NS
**n-6 PUFA**		
C18:2n-6 (Linoleic acid)	-.228	**.022**
C18:3n-6 (γ-Linolenic acid)	-.030	NS
C20:2n-6 (Eicosadienoic acid)	-.057	NS
C20:3n-6 (Dihomo-γ-linolenic acid)	.052	NS
C20:4n-6 (Arachidonic acid)	.119	NS
**n-3 PUFA**		
C18:3n-3 (Linolenic acid)	.152	NS
C20:5n-3 (Eicosapentaenoic acid)	-.099	NS
C22:5n-3 (Docosapentaenoic acid)	.095	NS
C22:6n-3 (Docosahexaenoic acid)	-.184	.066
Total Omega-3	-.121	NS
Omega-3 Index	-.167	.096

Associations between fatty acid species and age employing linear regression, have been represented. NS: non-significant ($p > .1$); SFA: saturated fatty acids; MUFA: monounsaturated fatty acids; PUFA: polyunsaturated fatty acids; omega 3 index: sum of eicosapentaenoic acid and docosahexaenoic acid, expressed as a percentage of total erythrocyte fatty acid measured.

Supplementary table S2: Comparison of erythrocyte fatty acid concentrations between males and females.

	**Males** (mean±SD)	**Females** (mean±SD)	**p**	**p[a]**	**p[b]**
SFA					
C14:0 (Myristic acid)	0.36 ± 0.06	0.40 ± 0.07	**.006**	**.003**	**.003**
C16:0 (Palmitic acid)	21.80 ± 1.56	21.86 ± 1.06	†.745	†.444	†.449
C18:0 (Stearic acid)	15.79 ± 1.26	15.84 ± 0.80	.812	.841	.664
C20:0 (Arachidic acid)	0.38 ± 0.07	0.42 ± 0.06	**.003**	**.034**	**.048**
C24:0 (Lignoceric acid)	4.98 ± 2.93	4.92 ± 1.20	.895	.919	.980
MUFA					
C14:1 (Myristoleic acid)	0.01 ± 0.00	0.01 ± 0.01	.685	.797	.713
C16:1 (Palmitoleic acid)	0.29 ± 0.10	0.34 ± 0.12	.074	**.032**	**.044**
C18:1n-9 (Oleic acid)	8.99 ± 0.66	8.64 ± 0.67	**.016**	.062	.051
C18:1n-7 (Vaccenic acid)	2.00 ± 0.55	1.92 ± 0.48	.493	.701	.824
C20:1n-9 (Eicosenoic acid)	0.27 ± 0.07	0.24 ± 0.06	.075	.283	.289
C24:1 (Nervonic acid)	4.89 ± 1.06	5.00 ± 0.83	.571	.567	.537
n-6 PUFA					
C18:2n-6 (Linoleic acid)	8.38 ± 1.44	8.72 ± 1.46	.280	.469	.443
C18:3n-6 (γ-Linolenic acid)	1.18 ± 1.63	0.74 ± 0.78	†.289	†**.038**	†.054
C20:2n-6 (Eicosadienoic acid)	0.14 ± 0.04	0.14 ± 0.41	.859	.636	.511
C20:3n-6 (Dihomo-γ-linolenic acid)	1.71 ± 0.41	1.58 ± 0.45	.200	.571	.435
C20:4n-6 (Arachidonic acid)	16.32 ± 2.44	15.94 ± 1.93	.399	.718	.618
n-3 PUFA					
C18:3n-3 (Linolenic acid)	0.14 ± 0.08	0.15 ± 0.10	.481	.569	.595
C20:5n-3 (Eicosapentaenoic acid)	1.46 ± 0.80	1.69 ± 0.91	.222	.456	.437
C22:5n-3 (Docosapentaenoic acid)	3.51 ± 0.70	3.15 ± 0.52	**.005**	**.009**	**.008**
C22:6n-3 (Docosahexaenoic acid)	7.30 ± 1.55	8.17 ± 1.45	**.008**	.060	**.048**
Total Omega-3	12.43 ± 2.52	13.18 ± 2.57	.172	.439	.422
Omega-3 Index	8.77 ± 2.10	9.86 ± 2.19	**.020**	.111	.096

Concentrations were compared between males (N=32) and females (N=68) wherein analyses were conducted using linear models with and without adjusting for covariates; p[a] indicates p values adjusted for neocortical amyloid load (NAL), age, *APOE ε4* status and years of education; p[b] indicates p values adjusted for NAL, age, *APOE ε4* status, years of education, body mass index (BMI), fatty acid supplement intake (cod liver oil, flaxseed oil), hormone replacement therapy. '†' indicates p-values obtained from variables transformed to the logarithmic scale for analyses. SFA: saturated fatty acids; MUFA: monounsaturated fatty acids; PUFA: polyunsaturated fatty acids; omega 3 index: sum of eicosapentaenoic acid and docosahexaenoic acid, expressed as a percentage of total fatty acids measured.

Supplementary table S3: Comparison of erythrocyte fatty acid concentrations between non-carriers and carriers of *APOE ε4* allele.

	**Non-carriers** (mean±SD)	***APOE ε4*** **carriers** (mean±SD)	**p**	$\mathbf{p^a}$	$\mathbf{p^b}$
SFA					
C14:0 (Myristic acid)	0.39 ± 0.07	0.38 ± 0.07	.534	.595	.680
C16:0 (Palmitic acid)	21.90 ± 1.31	21.62 ± 0.91	.363	.517	.638
C18:0 (Stearic acid)	15.80 ± 1.02	15.90 ± 0.76	.663	.441	.554
C20:0 (Arachidic acid)	0.41 ± 0.07	0.42 ± 0.07	.387	.785	.787
C24:0 (Lignoceric acid)	4.90 ± 2.10	5.10 ± 0.89	.663	.233	.278
MUFA					
C14:1 (Myristoleic acid)	0.01 ± 0.00	0.01 ± 0.00	.286	.493	.236
C16:1 (Palmitoleic acid)	0.32 ± 0.10	0.34 ± 0.16	.447	.158	.171
C18:1n-9 (Oleic acid)	8.75 ± 0.70	8.74 ± 0.64	.948	.736	.843
C18:1n-7 (Vaccenic acid)	1.92 ± 0.49	2.04 ± 0.55	.355	.342	.336
C20:1n-9 (Eicosenoic acid)	0.26 ± 0.06	0.24 ± 0.06	.194	.201	.188
C24:1 (Nervonic acid)	4.87 ± 0.88	5.33 ± 0.95	**.043**	**.025**	.051
n-6 PUFA					
C18:2n-6 (Linoleic acid)	8.83 ± 1.47	7.79 ± 1.06	**.003**	**.001**	**.003**
C18:3n-6 (γ-Linolenic acid)	0.84 ± 1.17	1.06 ± 1.00	.416	.353	.439
C20:2n-6 (Eicosadienoic acid)	0.15 ± 0.04	0.12 ± 0.03	**.016**	**.022**	**.023**
C20:3n-6 (Dihomo-γ-linolenic acid)	1.66 ± 0.44	1.49 ± 0.41	.136	**.038**	**.033**
C20:4n-6 (Arachidonic acid)	16.02 ± 2.17	16.20 ± 1.87	.723	.776	.812
n-3 PUFA					
C18:3n-3 (Linolenic acid)	0.16 ± 0.10	0.11 ± 0.07	**.049**	.176	.214
C20:5n-3 (Eicosapentaenoic acid)	1.59 ± 0.77	1.74 ± 1.24	†.746	†.609	†.569
C22:5n-3 (Docosapentaenoic acid)	3.28 ± 0.63	3.24 ± 0.51	.833	.335	.233
C22:6n-3 (Docosahexaenoic acid)	7.86 ± 1.46	8.01 ± 1.79	.685	.891	.959
Total Omega-3	12.73 ± 2.43	13.00 ± 3.10	.725	.557	.586
Omega-3 Index	9.45 ± 2.05	9.75 ± 2.79	.580	.620	.701

Levels were compared between *APOE ε4* non carriers (N=79) and carriers (N=21), wherein analyses were conducted using linear models with and without adjusting for covariates; p^a indicates p values adjusted for neocortical amyloid load (NAL), age, years of education and gender; p^b indicates p values adjusted for NAL, age, gender, BMI, years of education, fatty acid supplement intake (cod liver oil, flaxseed oil), hormone replacement therapy. '†' indicates p-values obtained from variables transformed to the logarithmic scale for analyses. SFA: saturated fatty acids; MUFA: monounsaturated fatty acids; PUFA: polyunsaturated fatty acids; omega 3 index: sum of eicosapentaenoic acid and docosahexaenoic acid, expressed as a percentage of total fatty acids measured.

Supplementary table S4: Comparison of erythrocyte fatty acid concentrations between non-memory complainers and memory complainers.

	**Non-complainers** (mean±SD)	**Memory complainers** (mean±SD)	**p**	**p[a]**	**p[b]**
SFA					
C14:0 (Myristic acid)	0.35 ± 0.06	0.40 ± 0.07	**.006**	**.017**	**.022**
C16:0 (Palmitic acid)	21.90 ± 1.57	21.83 ± 1.12	.810	.699	.590
C18:0 (Stearic acid)	15.66 ± 1.36	15.87 ± 0.81	.371	.302	.384
C20:0 (Arachidic acid)	0.38 ± 0.07	0.42 ± 0.06	**.037**	.061	.061
C24:0 (Lignoceric acid)	5.29 ± 3.30	4.83 ± 1.20	.311	.327	.378
MUFA					
C14:1 (Myristoleic acid)	0.014±0.006	0.016±0.009	.354	.331	.495
C16:1 (Palmitoleic acid)	0.32 ± 0.15	0.33 ± 0.11	.852	.768	.770
C18:1n-9 (Oleic acid)	8.88 ± 0.73	8.71 ± 0.67	.276	.433	.489
C18:1n-7 (Vaccenic acid)	1.87 ± 0.47	1.97 ± 0.51	.395	.311	.294
C20:1n-9 (Eicosenoic acid)	0.26 ± 0.07	0.25 ±0.06	.332	.449	.468
C24:1 (Nervonic acid)	4.89 ± 0.69	4.99 ± 0.97	.639	.747	.558
n-6 PUFA					
C18:2n-6 (Linoleic acid)	8.36 ± 1.75	8.70 ± 1.36	.320	.421	.541
C18:3n-6 (γ-Linolenic acid)	0.81 ± 0.77	0.91 ± 1.23	.707	.470	.377
C20:2n-6 (Eicosadienoic acid)	0.142 ±0.04	0.146 ±0.04	.702	.853	.859
C20:3n-6 (Dihomo-γ-linolenic acid)	1.62 ± 0.45	1.62 ± 0.44	.966	.805	.822
C20:4n-6 (Arachidonic acid)	16.32 ± 2.42	15.98 ± 2.00	.494	.629	.586
n-3 PUFA					
C18:3n-3 (Linolenic acid)	0.14 ± 0.08	0.15 ± 0.10	.584	.520	.586
C20:5n-3 (Eicosapentaenoic acid)	1.50 ± 0.81	1.66 ± 0.91	.437	.602	.608
C22:5n-3 (Docosapentaenoic acid)	3.43 ± 0.80	3.22 ± 0.52	.146	.309	.234
C22:6n-3 (Docosahexaenoic acid)	7.78 ± 1.79	7.93 ± 1.45	.690	.938	.951
Total Omega-3	12.86 ± 2.65	12.97 ± 2.56	.852	.943	.972
Omega-3 Index	9.28 ± 2.28	9.59 ± 2.20	.560	.876	.804

Levels were compared between non-complainers (N=24) and memory complainers (N=76), wherein analyses were conducted using linear models with and without adjusting for covariates; p^a indicates p values adjusted for neocortical amyloid load (NAL), age, years of education and gender; p^b indicates p values adjusted for NAL, age, gender, BMI, years of education, fatty acid supplement intake (cod liver oil, flaxseed oil), hormone replacement therapy. SFA: saturated fatty acids; MUFA: monounsaturated fatty acids; PUFA: polyunsaturated fatty acids; omega 3 index: sum of eicosapentaenoic acid and docosahexaenoic acid, expressed as a percentage of total fatty acids measured. Memory Assessment Clinic-Questionnaire (MAC-Q) scores were employed to categorise participants into non-complainers (MAC-Q<25) and memory complainers (MAC-Q≥25).

Supplementary table S5: Comparison of dietary intake of fatty acids altered between high and low NAL and, non-carriers and carriers of *APOE ε4*, males and females, and memory complainers and non-complainers.

**a.**	**Low NAL**	**High NAL**	**p**	**p[a]**
C20:4n-6 (Arachidonic acid)	0.057±0.02	0.060±0.02	.581	.270
C22:5n-3 (Docosapentaenoic acid)	0.040±0.02	0.042±0.02	.774	.390
**b.**	**Non- ε4**	***APOE* ε4**		
C18:2n-6 (Linoleic acid)	8.61±3.49	8.46±3.60	.866	.769
C20:2n-6 (Eicosadienoic acid)	0.015±0.013	0.009±0.006	†.051	†.063
C20:3n-6 (Dihomo-γ-linolenic acid)	0.008±0.005	0.006±0.003	.040	.024
**c.**	**Males**	**Females**		
C14:0 (Myristic acid)	3.06±1.14	2.69±1.16	.145	.162[b]
C16:1 (Palmitoleic acid)	1.60±0.46	1.40±0.60	.098	.132 [b]
C20:0 (Arachidic acid)	0.25±0.15	0.23±0.12	.484	.455 [b]
C22:6n-3 (Docosahexaenoic acid)	0.22±0.14	0.23±0.18	.828	.833 [b]
C22:5n-3 (Docosapentaenoic acid)	0.04±0.02	0.04±0.02	.764	.739 [b]
**d.**	**Memory complainers**	**Non-complainers**		
C14:0 (Myristic acid)	2.61±1.10	2.87±1.18	.344	.125

Comparison of the dietary intake of significantly altered erythrocyte fatty acids between participants with (a) high neocortical amyloid load (NAL, n=35) and low NAL (n=65), (b) *APOE ε4* non-carriers (n=79) and carriers (n=21), (c) males (n=32) and females (n=68), and (d) memory complainers (n=76) and non-complainers (n=24), based on a MAC-Q score cut-off of 25. Analyses were conducted using linear models with and without adjusting for covariates; p[a] indicates p values adjusted for age, body mass index, years of education and gender; [b] indicates p values adjusted for age, body mass index and years of education. '†' indicates p-values obtained from variables transformed to the logarithmic scale for analyses. Dietary fatty acid concentrations were calculated in grams/day employing the Cancer Council of Victoria food frequency questionnaire. Fatty acid intakes have been represented as mean±SD.

Reference:

[1] Nasreddine ZS, Phillips NA, Bedirian V, Charbonneau S, Whitehead V, Collin I, et al. The Montreal Cognitive Assessment, MoCA: a brief screening tool for mild cognitive impairment. J Am Geriatr Soc. 2005;53:695-9.
